# Dethiothermospora halolimnae gen. nov., sp. nov., a novel moderately halophilic, thermotolerant, bacterium isolated from a brine lake

**DOI:** 10.1099/ijsem.0.006760

**Published:** 2025-04-30

**Authors:** Luke A. Fisher, Saloni R. Dangre, Arthur Odenheimer, Nirav Patel, Peter T. Doran, Jeff S. Bowman, Britney E. Schmidt, Douglas H. Bartlett

**Affiliations:** 1 Scripps Institution of Oceanography, University of California San Diego, La Jolla, CA, USA; 2 University of California Los Angeles, Los Angeles, CA, USA; 3 Louisiana State University, Baton Rouge, LA, USA; 4 Cornell University, Ithaca, NY, USA

**Keywords:** anaerobes, Brine lakes, *Dethiothermospora halolimnae*, *Thermohalobacteraceae*, Western Australia transient lakes

## Abstract

A novel, strictly anaerobic, slightly alkaliphilic, halotolerant, peptide- and amino acid-utilizing bacterial strain, SD1^T^, was isolated from a hypersaline lake in Western Australia. The strain stained Gram-negative and was a motile, spore-forming rod. The strain grew between 15 and 50 °C (optimum 40 °C), 1–15% w/v sodium chloride (optimum 5%) and pH 6.0–10.0 (optimum 9.0). Major fatty acids included anteiso-C15 : 0 (24.9%), C14 : 0 dimethyl acetyl (13.2%), anteiso-C15 : 0 dimethyl acetyl (11.5%) and iso-C15 : 0 (10.4%). The DNA G+C content was 30.3 mol%. The isolate did not grow using any tested sugars but grew well on arginine and glycine. It is capable of using elemental sulfur and thiosulfate as alternate electron acceptors, but not sulfide, sulfate, nitrate or nitrite. 16S rRNA gene similarity indicates that the isolate is related to *Sporosalibacterium tautonense* MRo-4^T^ (94.33% identity). SD1^T^ showed 76.18%–76.31% average nucleotide identity with other strains within the family *Thermohalobacteraceae*. Phylogenetics, based on the 16S rRNA gene and whole-genome sequence, as well as phenotypic analysis, differentiates the isolate from close neighbors. We propose that SD1^T^ represents a novel species in a new genus, which we have named *Dethiothermospora halolimnae* gen. nov., sp. nov., type strain SD1^T^ (DSM 117405^T^ = TSD-443^T^). From this work, we also propose repositioning of the genus *Anaeromonas* to the family *Thermohalobacteraceae*.

## Data availability

The whole-genome sequence and 16S rRNA gene sequence of the type strain SD1^T^ (DSM 117405^T^ = TSD-443^T^) are available on National Center for Biotechnology Information (NCBI) under the GenBank and sequence accession numbers GCA_037577855.1 and PP133767.1, respectively. Supplementary files S1 and S2 show the calculated average amino acid identity and percent of conserved proteins, respectively, of SD1^T^ with neighbors. These files are available in the online version of this article. 16S amplicon sequencing data from the lake from which SD1^T^ was isolated, as well as from other nearby lakes, will be published in a forthcoming manuscript but are available upon request under the NCBI BioProject PRJNA1244000.

## Introduction

Within the Yilgarn Craton in the southern portion of Western Australia, there exist thousands of shallow ephemeral saline lakes. These lakes vary significantly in pH and salinity, with some reaching pH values as low as 1.7 and total dissolved solids (TDS) up to 320 g l^–1^, while others are nearly fresh with neutral to basic pH [1,3]. Winter rains and hot, dry summers drive significant seasonal variations in lake geochemistry, commonly leading to complete evaporation and salt encrustation [4]. These rare, easily accessible acid brines are of great interest to the field of astrobiology as they provide excellent analogs to hypothesized ancient brine environments on Mars [5]. Furthermore, the combination of stressors in these saline lakes makes for unique polyextreme environments to study the geochemical window for extremophilic life on Earth. To the best of our knowledge, there is no published taxonomic information about microbial communities in our study site (Lake Koorkoordine) nor cultured isolates. Porewater samples from this lake were inoculated into four media types of varying pH and salinity to isolate members of diverse taxa. Here, we describe a novel isolate in the phylum *Bacillota* in the class *Tissierellia* within the order *Tissierellales* in the family *Thermohalobacteraceae*.

At the time of writing, the family *Thermohalobacteraceae* contains seven genera. Members of this family mainly occur in anoxic sediment, are strictly anaerobic, are Gram-stain negative or positive and produce spores. Cells utilize carbohydrates, peptides and amino acids via fermentation and are slender motile rods that are moderately thermo/halophilic. G+C content is typically low (~28–33 mol%), and fatty acids iso-C15 : 0 and iso-C15 : 0 DMA (dimethyl acetyl) are dominant [6]. Microbes in this family are ubiquitous and are found in marine sediments, salterns, microbial mats and subsurface environments [7,13].

Here, we isolate and characterize a novel species of a novel genus in the family *Thermohalobacteraceae,* which is a spore-forming, slightly alkaliphilic, moderately halophilic, thermotolerant, strictly anaerobic heterotroph isolated from Lake Koorkoordine in the Yilgarn Craton, Western Australia.

## Collection2

Porewater samples were collected on 7 August 2022, at the southern portion of Lake Koorkoordine in Western Australia (31.18128 °S, 119.3123 °E). At the time of collection, the pH and temperature of the porewater were 6.5 and 10.3 °C, respectively, had a water activity (*a*_w_) of 0.74 and TDS of 320.06 g L^–1^. Temperature and pH were measured *in situ* using a HOBO^®^ pH and Temperature Data Logger, *a*_w_ of porewater was measured using an AQUALAB^®^ 4TE water activity meter, and TDS was determined via gravimetric analysis. To collect porewater, sippers were made in the laboratory by heat sealing the tip of a plastic 10 ml serological pipette over a Bunsen burner. Small holes were made near the sealed tip using a hot 23 G needle followed by autoclaving. The sipper was inserted into shoreline sediment to a depth of 14 cm, and porewater was drawn using a sterile 30 ml syringe connected by sterile flexible plastic tubing. All components were flushed in the field with Ultra High Purity (UHP) nitrogen gas for 1 min prior to use. Anaerobic porewater was immediately inoculated (1 : 50 dilution) in the field into serum vials containing anaerobic 50 g l^–1^ or 232 g l^–1^ NaCl, DSMZ 479 medium containing per litre (1.5 g KCl, 6.0 g MgCl_2_•6H_2_O, 0.4 g CaCl_2_•2H_2_O, 1.0 g NH_4_Cl, 0.3 g K_2_HPO_4_, 2 g yeast extract, 2 g trypticase peptone, 0.5 ml 0.1% Na-resazurin solution, 1.5 g Na_2_CO_3_, 2.0 g trimethylamine-HCl, 0.2 g 2-mercaptoethanesulfonic acid (coenzyme M), 0.25 g Na_2_S•9H_2_O, 10 ml modified Wolin’s mineral solution) amended with 10 ml l^–1^ American Type Culture Collection (ATCC^®^) MD-VS^™^ vitamin solution at varying pH ranges from 2 to 10 [14]. After inoculation, vials were flushed with UHP nitrogen gas for 1 min using 23 G needles. No traces of oxygen were observed (as indicated by the lack of pink resazurin coloration) at any point during inoculation or downstream analysis. Enrichments were incubated in the dark at room temperature without shaking for 1 month.

## Isolation

The anaerobic technique of Hungate was used throughout isolation and characterization [15]. Enrichments were serially diluted into 50 g l^–1^ NaCl pH 7.0 DSMZ 479 medium (recipe above) containing 10 ml l^–1^ ATCC^®^ MD-VS^™^ vitamins at varying pHs. Cultures were incubated in the dark at room temperature without shaking. Growth was observed visually within a week and was confirmed via standard light microscopy and PCR. One milliliter of culture was centrifuged, washed with 1 ml sterile dH_2_O, concentrated in 40 µl of nuclease-free water and boiled at 95 °C for 10 min. The boiled-prepped sample was used as a template for PCR with the universal bacterial primers 27F and 1492R [16]. Sanger sequencing results from clean amplicons indicated the absence of methanogens in our cultures (the intended target of our original study), so coenzyme-M, Na_2_CO_3_ and trimethylamine-HCl were omitted in subsequent cultures. This new medium is hereby referred to as ‘DL’ (d-type low salt) medium. Cultures with visual growth and cleaner Sanger sequences were diluted to extinction for six more rounds in DL medium at pH 5.0, and cultures were finally purified after repeated use of the Hungate Roll tube technique [17]. A single colony isolate (1–3 mm) from 10-fold serially diluted cultures was inoculated into fresh pH 7.0 DL medium and allowed to grow without shaking at 30 °C until visual growth was observed after about 2 days. This process was repeated at least twice before the culture was considered pure and was then preserved in 25% (v/v) glycerol at −80 °C. The strain SD1^T^, the focus of this work, was used for further study and characterization.

## Physiology and chemotaxonomy

Fresh mid-upper log growth phase SD1^T^ cultures were prepared from frozen stock and diluted 1 : 100 into triplicate anaerobic DL medium in Hungate tubes at pH 3–7 in intervals of 1.0, 7.5–10 in intervals of 0.5 and pH 11. Cultures were then incubated at 25 °C with shaking. MES buffer was added at pH 6.0, HEPES at pH 7–8 and NaOH-glycine at pH 9–10 to a final concentration of 10 mM for all buffers. Growth was evaluated at 600 nm using a Thermo Scientific™ Spectronic 20^+^ spectrophotometer. SD1^T^ exhibited growth from pH 6 to 10 with optimal growth at pH 9.0 (Table 1). Temperature optima of SD1^T^ were assessed as described above in pH 9.0 DL medium. Growth occurred between 15 and 50 °C with an optimal temperature of 40 °C. This optimum is consistent with closely related species; however, SD1^T^ has the broadest temperature range for growth (Table 1).

**Table 1.** Characteristics of strain SD1^T^ (1) and closely related strains, *Sporosalibacterium faouarense* SOL3f37^T^ (2), *S. tautonense* MRo-4^T^ (3), *Clostridiisalibacter paucivorans* 37HS60^T^ (4) and *Abyssisolibacter fermentans* MCWD3^T^ (5). ND, no data available; w+, weakly positive growth. Compared to four close neighbors, SD1^T^ can use thiosulfate and elemental sulfur as alternate electron acceptors and has the broadest temperature ranges for growth and the highest pH optima. Data obtained from [9,11, 31, 32]

Characteristic	1	2	3	4	5
Origin	Porewater from Lake Koorkoordine, Western Australia	Hydrocarbon-polluted soil, El Faouar area, Tunisia	Microbial mat, TauTona gold mine, South Africa	Olive mill wastewater, Morocco	Deep sea sediment, Ulleung Basin, Sea of Japan
Minimum/optimum/maximum temperature (°C) for growth	15/40/50	20/40/48	25/42/50	20/42/50	15/29/40
Minimum/optimum/maximum pH for growth	6.0/9.0/10.0	6.2/6.9/8.1	7.0/7.4–7.9/8.8	5.5/6.8/8.5	5.0/6.5/9.0
Minimum/optimum/maximum NaCl(% w/v)	1/5/15	0.05/4/15	0.5/2/10	1/5/10	1/2/5
Requirement of yeast extract for growth (g l^–1^):	0.1	2	0.1	0.1	0.5
Reduction of S^0^:	**+**	**–**	**–**	**–**	**–**
Reduction of SO_4_^2^−:	**–**	**–**	**–**	**–**	**–**
Reduction of S_2_O_2_^−3^:	**+**	**–**	**–**	**–**	**–**
Reduction of NO_3_^–^:	**–**	**–**	**–**	**–**	**–**
Reduction of NO_2_^–^:	**–**	**–**	**–**	**–**	**–**
DNA G+C content (mol%):	30.3	30.7	32.9	33.0	28.8
Growth on substrates:					
Arginine	**+**	**–**	**–**	**–**	**–**
Glycine	**+**	**–**	**–**	**–**	**–**
Cysteine	**–**	**–**	**–**	**+**	**–**
Serine	**–**	**–**	**–**	**+**	**–**
Lysine	**–**	**–**	**–**	**+**	**–**
Valine	**–**	**–**	**–**	**+**	**–**
Fructose	**–**	**+**	**+**	**–**	**+**
Galactose	**–**	**–**	**–**	**–**	**–**
Glucose	**–**	**+**	**–**	**–**	**–**
Glycerol	**–**	**–**	**–**	**–**	**–**
Mannitol	**–**	**+**	w+	**–**	**–**
Fumarate	**–**	**+**	w+	**+**	**–**
Cellobiose	**–**	**–**	**+**	**–**	**+**
Cellulose	**–**	**–**	w+	**–**	nd
Chitin	nd	nd	w+	nd	nd
Xanthan gum	**–**	nd	w+	nd	nd

**Fig. 1.**
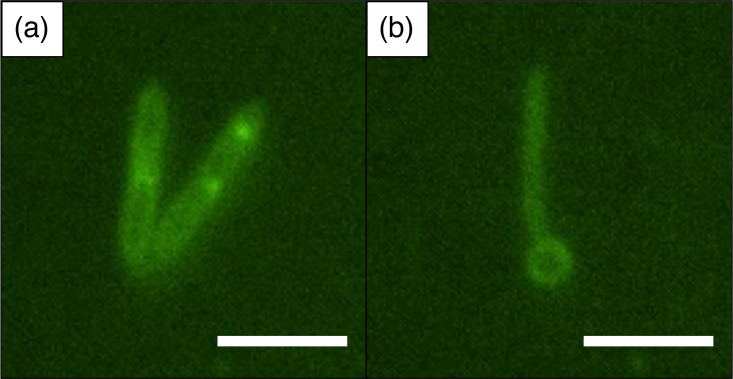
Micrographs of fresh (**a**) and 6 day old (**b**) SD1^T^ cells stained with acridine orange at 1000x magnification (500 nm Ex./526 nm Em.). Bright spots (**a**) were common among cells. Terminal spores shown in panel B were common in older or nutrient-limited cultures. Scale bars are 5 µm.

Cell morphology was examined using a Nikon^®^ Eclipse TI microscope at 600× magnification and a Bruker^®^ Dimension Fast Scan atomic force microscope (AFM). Fresh cultures were fixed and stained with acridine orange (500 nm Ex./526 nm Em.) for fluorescence microscopy. Cells were straight, slender, small rods (0.5×0.5–6 µm) that grew in pairs or singly (Fig. 1a). Older cultures developed terminal spores (Fig. 1b). Flagella were not observed via fluorescence microscopy or AFM, but cells were motile as verified by stab inoculations into DL soft agar medium (0.3% agar w/v) and allowing for growth at 30 °C for 3 days. A total of 70 features related to flagella biosynthesis and function were found in the genome of SD1^T^. SD1^T^ stained Gram-negative.

To assess the use of various carbon sources, SD1^T^ was grown in pH 9.0 basal medium containing per liter distilled water: 7.5 g KCl, 30 g MgCl_2_•6H_2_O, 2 g CaCl_2_•2H_2_O, 5 g NH_4_Cl, 2 g K_2_HPO_4_•3H_2_O, 0.5 g cysteine-HCl, 10 ml ATCC^®^ MD-VS vitamins, 10 ml modified Wolin’s mineral solution and 2.5 ml of 5% Na_2_S•9H_2_O. Vitamins and sodium sulfide were added as sterile anoxic stock solutions after autoclaving. The basal medium was then amended with yeast extract as a sterile anoxic stock solution to a final concentration of 0.1, 0.2 or 2 g l^–1^. Anoxic sterile stock solutions of substrates were added to a final concentration of 20 mM.

Alternate electron acceptor tests were carried out in triplicate Hungate tubes and determined colorimetrically using the Cline reaction for sulfur tests and Quantofix^®^ test strips for nitrate/nitrite reduction [18]. Nitrate (20 mM), nitrite (2 mM), thiosulfate (20 mM), elemental sulfur (~0.1% w/v) and sulfate (20 mM) were added as sterile anoxic stock solutions, except for elemental sulfur, which was added as a sterile paste, to basal pH 9.0 medium at a yeast extract concentration of 0.2 g l^–1^. Sodium sulfide was omitted from the basal medium for all alternate electron acceptor tests. All fatty acid methyl ester analyses and identification were carried out by DSMZ Services, Leibniz-Institut DSMZ–Deutsche Sammlung von Mikroorganismen und Zellkulturen GmbH, Braunschweig, Germany. Ten fatty acids were detected at levels above 2% in strain SD1^T^: anteiso-C15 : 0 (24.9%), C14 : 0 DMA (13.2%), anteiso-C15 : 0 DMA (11.5%), iso-C15 : 0 (10.4%), C14 : 0 (7.4%), C14 : 0 aldehyde (ALDE) (7.2%), iso-C15 : 0 DMA (6.0%), iso-C14 : 0 (4%), C16 : 0 DMA (2.4%) and iso-C15 : 0 ALDE (2.4%) (Table 2).

**Table 2.** Fatty acid profile of SD1^T^ (1) compared to *Sporosalibacterium faouarense* SOL3f37^T^ (2), *S. tautonense* MRo-4^T^ (3), *Clostridiisalibacter paucivorans* 37HS60^T^ (4) and *Abyssisolibacter fermentans* MCWD3^T^ (5). Data shown are percentages of the total fatty acids derived from peak areas in gas chromatography plots greater than or equal to 0.5%

Fatty acids	1*	2*	3*	4*	**5†**
**Saturated:**					
C14 : 0	7.4	2.0	5.3	14.4	2.6
C15 : 0			0.8		5.0
C16 : 0	0.9	0.9	5.3	3.2	1.6
C18 : 0	0.5	0.9	3.8	0.9	
**Saturated and branched:**					
iso-C13 : 0		4.5	2.5		5.4
anteiso-C13 : 0	0.6	1.4			
iso-C14 : 0	4.0	1.2	0.5	1	
iso-C15 : 0	10.4	37.5	39.8	17.3	30.0
anteiso-C15 : 0	24.9	5.4	2.9	3.9	7.6
**Dimethyl acetyl (DMA) forms:**					
iso-C13 : 0 DMA		1.2	0.5		
C14 : 0 DMA	13.2	1.7	1.5	3.6	3.9
iso-C15 : 0 DMA	6.0	21	7.8	1.7	16.9
anteiso-C15 : 0 DMA	11.5	1.9	0.5		2.7
C16 : 0 DMA	2.4	1.6	2.9	18.8	5.3
iso-C16 : 0 DMA	1.3			0.7	
iso-C17 : 0 DMA		0.6		2.4	
C18 : 0 DMA		0.5		1.0	
**Aldehyde (ALDE) forms:**					
C14 : 0 ALDE	7.2	0.9	0.6	2.1	
iso-C15 : 0 ALDE	2.4	9.0	2.7	0.8	
C16 : 0 ALDE	0.7		0.6	6.4	
**Unsaturated:**					
C14 : 1 ω7c				0.9	
C15 : 1 ω7c			4.1		
iso-C15 : 1 ω7c		1.3		2.1	
C16 : 1 ω7/9 c			2.8	2.0	
C16 : 1 ω7/9 c DMA			0.7	4.0	
C16 : 1 ω7c ALDE				2.2	
C17 : 1 ω9c DMA			0.5	1.8	
C18 : 1 ω9c			3.4	0.5	
C18 : 1 DMA				1.8	
Summed feature: C18 : 11ωt or cis-C18 : 1 ω11c			1.8		
**Other:**					
Sum^1^ (C15 : 0 DMA and/or C14 : 0 3OH)					9.7

*Data obtained from this study.

†data obtained from [11].

SD1^T^ was able to grow on arginine and glycine, but not on cysteine, serine, lysine, valine, fructose, galactose, glucose, glycerol, mannitol, fumarate, cellobiose, cellulose or xanthan gum (Table 1). SD1^T^ was able to use both elemental sulfur and thiosulfate as alternate electron acceptors but was unable to use sulfate, sulfide, nitrite or nitrate. SD1^T^ was determined to be a strict anaerobe, as it did not grow in medium containing trace amounts of oxygen, as indicated by the pink coloration of sodium resazurin. SD1^T^ had a broad temperature range for growth (15–50 °C) and grew optimally at pH 9.0, making it slightly alkaliphilic, whereas close relatives grow best in near neutral pH ranges of 6.5–7.9 (Table 1). The predominant fatty acids of SD1^T^ were anteiso-C15 : 0 (24.9%), C14 : 0 DMA (13.2%), anteiso-C15 : 0 DMA (11.5%) and iso-C15 : 0 (10.4%).

## Phylogenetic analysis and genome features

**Fig. 2.**
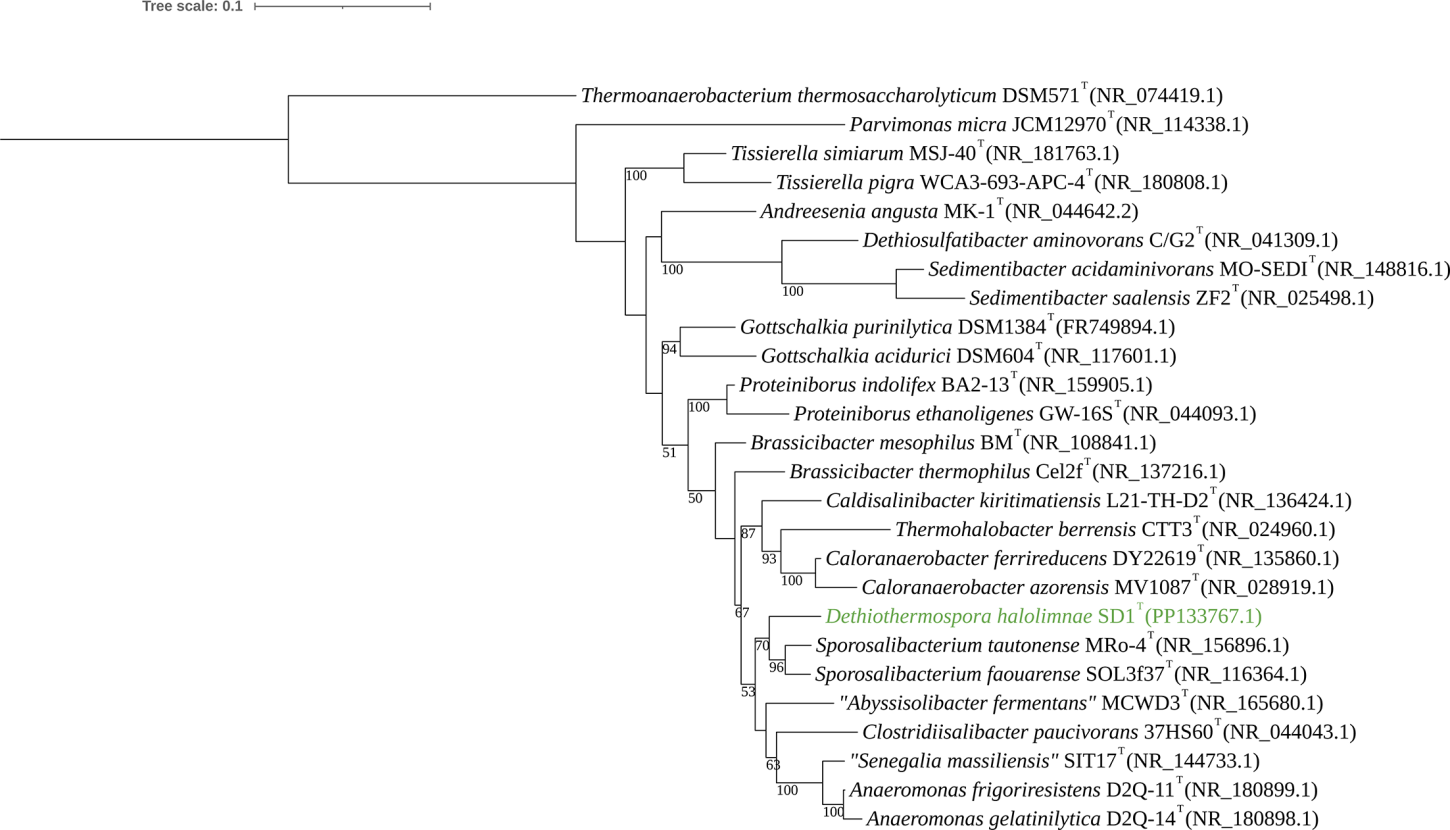
Phylogenetic tree based on the 16S rRNA gene sequence of SD1^T^ (green) and close neighbors with *Thermoanaerobacterium thermosaccharolyticum* DSM 571 (accession no. NR_074419.1) as an outgroup. 16S rRNA sequences were downloaded from NCBI, aligned with MAFFT v7.551 and trimmed using gBlocks v0.91b. The phylogenetic tree was constructed from the trimmed alignment using IQ-TREE v2.3.6 using the GTR+G model. Bootstrap values (expressed as a fraction of 1,000 replicates) equal to or greater than 50 are indicated at nodes. The tree was configured using iTOL (Interactive Tree of Life v7), and scale refers to the number of nucleotide substitutions per site between taxa.

DNA was extracted from fresh SD1^T^ culture using the NEB^®^ Monarch gDNA kit and sequenced on an Illumina platform at the UC San Diego Microbiome Core Facility. Sequences were trimmed, assembled and annotated using Trimmomatic v0.36, PRINSEQ v0.20.4, SPAdes v3.15.3 and Prokka v1.14.5 on Kbase [19,22]. The G+C content of the draft genome of SD1^T^ was 30.3 mol%. The accession number for the draft genome sequence (GenBank) is GCA_037577855.1. The 16S rRNA gene sequence (accession no. PP133767.1) was initially amplified directly from purified genomic DNA from the isolate using the universal bacterial primers 27F and 1492R, and the purified PCR product was sequenced at Retrogen. Publicly available 16S rRNA sequences of closely related species were downloaded from NCBI, aligned with MAFFT v7.551 [23], and trimmed with GBlocks v0.91.1 [24]. A 16S rRNA gene tree of SD1^T^ was made using IQ-TREE v2.3.6 using the GTR+G model with 1,000 bootstrap replicates [25,27]. To verify the strain’s phylogeny, additional 16S rRNA gene trees were constructed via maximum likelihood using RAxML v8.2.12 [28], nearest neighbor interchanges using FastME v2.1.6.1.1 [29] and maximum parsimony using TNT v2.1.6.1.1 [30]. In all cases, 16S rRNA gene analysis revealed that SD1^T^ is a member of the family *Thermohalobacteraceae* within the order *Tissierellales* in the class *Tissierellia,* forming a separate branch with members of *Sporosalibacterium* (Figs 2 and S1–S3). Sequence alignment to NCBI’s 16S rRNA database indicated *Sporosalibacterium tautonense* MRo-4^T^ (94.33% identity), *S. faourense* SOL3f37^T^ (94.19% identity) and *Clostridiisalibacter paucivorans* 37HS60^T^ (93.18% identity) were the closest relatives [31,33]. 16S rRNA gene sequence identity values of these close neighbors all fall below the revisited genus level threshold of 94.5% similarity [34]. Based on 16S amplicon sequencing of lake material from Koorkoordine and other nearby lakes within the Yilgarn Craton, SD1^T^ is quite rare, representing less than 0.01% of sequencing reads when detected. From 217 DNA samples extracted from brine, sediment, porewater, evaporite and biofilms across 35 lakes, only 24 out of 2.3 million quality-filtered 16S reads had a 16S rRNA gene sequence similarity ≥99.5% to that of strain SD1^T^ (35; Klempay and Bowman, unpublished results). blast results of the 16S rRNA gene of SD1^T^ revealed high percent identity matches (≥98.61%) associated with only two uncultured organism clones from hypersaline microbial mats [36,37]. These close relatives were also in very low abundance based on these studies.

**Fig. 3.**
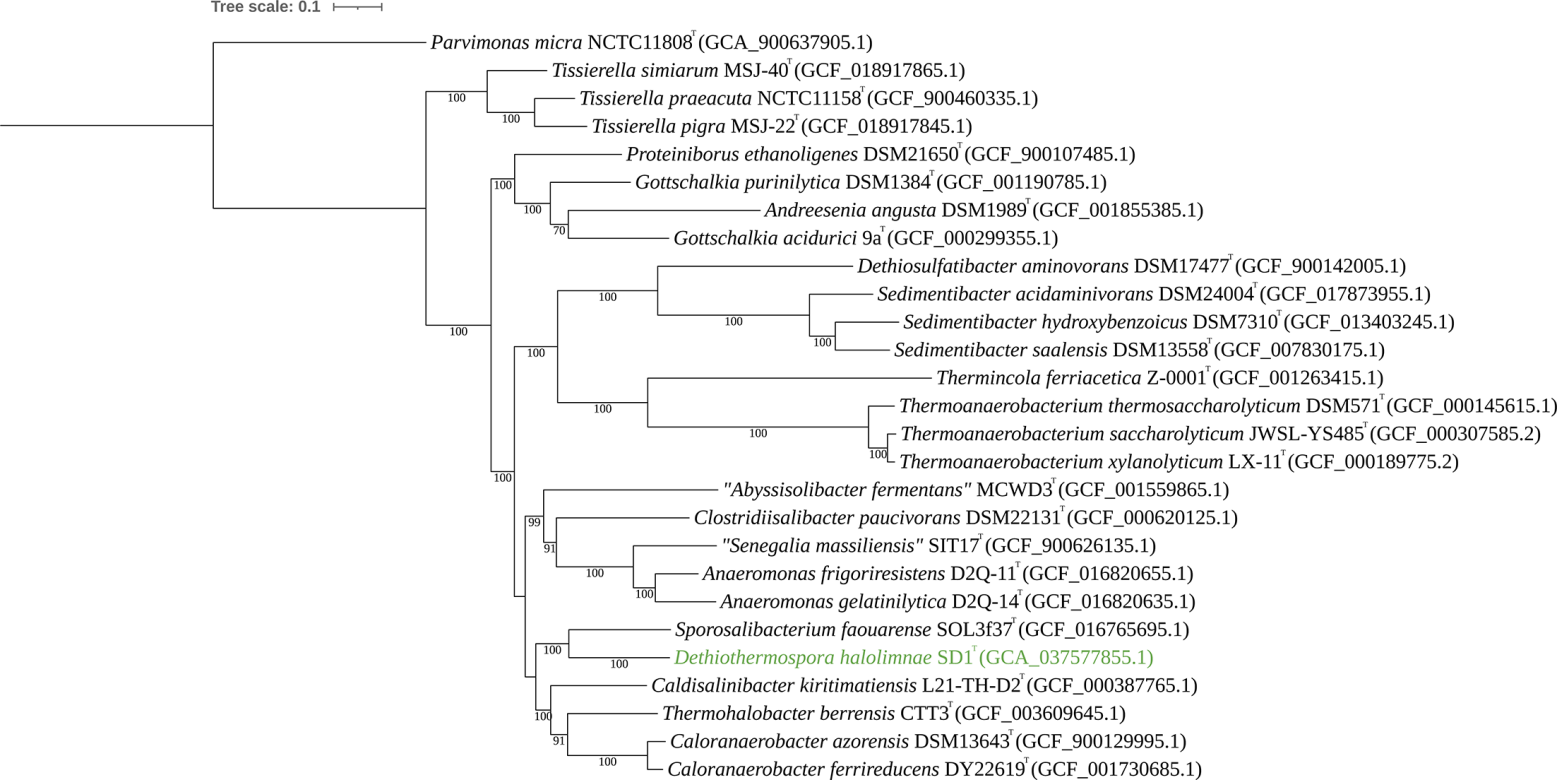
A phylogenomic tree of SD1^T^ in comparison to its close neighbors based on 119 core proteins constructed using GToTree v1.8.6. *Parvimonas micra* NCTC 11808^T^ (GCA_900637905.1) was used as an outgroup. A text file containing NCBI accession numbers was used as input using the HMM ‘Firmicutes’ setting with 119 single marker protein targets and inferred using IQ-TREE with ultrafast-bootstrap support. Bootstrap values (expressed as a fraction of 1,000 replicates) greater than or equal to 70 are indicated at nodes. The tree was configured on iTOL, and the scale refers to the number of amino acid substitutions per site between taxa.

To better understand the phylogeny of SD1^T^, the whole-genome sequence was investigated. The SD1^T^ genome was analysed using the Classify Microbes with GTDB-Tk v2.3.2 app within KBase, which compares user input genomes to the Genome Taxonomy Database (GTDB) using a FastTree inferred method from 120 single-copy marker proteins [38]. Using this approach, SD1^T^ was assigned at the family level to *Thermohalobacteraceae* with a relative evolutionary divergence value of 0.759, indicating it likely represents a novel species within a novel genus [38]. Further analysis was conducted using GToTree v1.8.6 using the pre-packaged single-copy marker protein set for ‘Firmicutes’ (119 gene targets) [39,45]. In short, gene prediction on input genomes in FASTA format was performed using Prodigal v2.6.3. Target genes were identified via HMMER3 v3.2.2, aligned individually using muscle v5.1, trimmed with TrimAl v1.4 and concatenated before phylogenetic analysis with IQ-TREE. TaxonKit was employed to link taxonomic IDs to complete lineages. This tree also placed SD1^T^ within the family *Thermohalobacteraceae,* with its closest neighbor being *S. faourense* SOL3f37^T^ (Fig. 3). Another bacterial phylogenomic tree was constructed using the Insert Genome into Species Tree v2.20 application on KBase, which generates a phylogenetic tree of user input genomes to public RefSeq genomes using 49 core universal genes defined by Clusters of Orthologous Groups gene families [44]. This tree also indicated that SD1^T^ is most closely related to *S. faourense* SOL3f37^T^ (Fig. S4). Average nucleotide identity (ANI) comparison between the genomes of *Dethiothermospora halolimnae* SD1^T^ and *S. faourense* SOL3f37^T^ was performed using FastANI [46], yielding a value of 76.31%. As a comparison of ANI across genera within the family *Thermohalobacteraceae,* the closely related species *S. faourense* SOL3f37^T^ and *C. paucivorans* 37HS60^T^ were compared, giving a value of 76.08%. Other close neighbors of SD1^T^ were also compared, yielding values ranging from 75.68% to 76.18%. These ANI values were significantly below the 95–96% prokaryotic species delineation boundary [47,49]. A visualization of these pairwise ANI comparisons can be found in Fig. S5. Average amino acid identity (AAI) was calculated from downloaded publicly available genomes between members of *Thermohalobacteraceae* using EzAAI v1.2.3 (File S1 [50]). AAI values of SD1^T^ compared to other currently accepted members of *Thermohalobacteraceae* based on Spring [6] ranged from 62.15% when compared to *Senegalia massiliensis* to 66.39% when compared to *S. faourense* SOL3f37^T^. AAI was as high as 69.05% across different genera within *Thermohalobacteraceae*. AAI values of SD1^T^ compared to other close neighbors are within the generally accepted genus demarcation threshold of 65–72% [51]. Percent of conserved protein (POCP) was calculated using POCP-nf [52] to compare the genome of SD1^T^ to other close neighbors (File S2). POCP ranged from 46.56% when compared to *C. paucivorans* 37HS60^T^ and 54.17% when compared to *S. faourense* SOL3f37^T^. POCP values across genera within *Thermohalobacteraceae* were as high as 65.39%. Although POCP values of SD1^T^ compared to *S. faourense* SOL3f37^T^ were 54.17%, slightly above the arbitrary 50% cutoff [53], we still find it appropriate to place SD1^T^ in a novel genus given that POCP values between other genera within *Thermohalobacteraceae* were as high as 65.39%.

Based on 16S rRNA gene phylogeny, phylogenomics, phenotypic and chemotaxonomic analysis, we find that SD1^T^ is distinct from the members of *Thermohalobacteraceae,* representing a novel species within a novel genus which we have named *D. halolimnae*. It is important to note that with the ongoing flux of taxonomic rearrangements based on new phylogenomic analyses in the GTDB framework, some genera previously placed in the family *Thermohalobacteraceae* are now mono-generic (containing only one genus) family lineages [54,55]. The resolution of the current differences in family-level associations between the NCBI and GTDB databases will require future higher level taxonomic analyses.

## Repositioning of the genus *Anaeromonas*

Based on NCBI taxonomy, our analyses indicate that the genus *Anaeromonas* [56], currently containing the two characterized strains *Anaeromonas frigoriresistens* DSQ-11^T^ and *Anaeromonas gelatinilytica* D2Q-14^T^, should be moved from the order *Eubacteriales* within the class *Clostridia* to the family *Thermohalobacteraceae* within the order *Tissierellales* within the class *Tissierellia*. Based on phylogenomic analysis and 16S rRNA gene trees, *Anaeromonas* forms a robust clade (bootstrap values of 100) with *C. paucivorans* 37HS60^T^ and *S. massiliensis* SIT17, both members of the family *Thermohalobacteraceae* [6], and *Abyssisolibacter fermentans* MCWD3^T^ (Figs2^3^ and S1–S4). When compared to other members of *Thermohalobacteraceae*, POCP of *A. frigoriresistens* DSQ-11^T^ ranged from 48.11% to 64.88%, and AAI ranged from 63.04% to 72.06% (Files S1 and S2). These values are similar to other genus demarcations within this family, and based on these two metrics, *Anaeromonas* grouped more closely at the family level with *Thermohalobacteraceae* compared to other close families.

We also found that based on phylogenomics using GToTree, *A. fermentans* MCWD3^T^ forms a strong clade (bootstrap values of 100) with *S. massiliensis* and *Anaeromonas* spp., suggesting that its placement within *Thermohalobacteraceae. A. fermentans* MCWD3^T^ is phenotypically similar to other members within this family and, based on 16S rRNA gene alignment using NCBI’s 16S rRNA database, groups most closely to *S. tautonense* MRo-4^T^ (94.42%), *S. faourense* SOL3f37^T^ (94.25%), and *D. halolimnae* SD1^T^ (93.54%). However, due to the low POCP and ANI values of MCWD3^T^ when compared to members of *Thermohalobacteraceae,* we suggest that *A. fermentans* may represent a separate family lineage within the class *Tissierellia* (Files S1 and S2), but future study is necessary to clarify the strain’s phylogeny.

## Description of *Dethiothermospora* gen. nov.

*Dethiothermospora*: (De.thi.o.ther.mo.spo’ra. L. pref. *de*, from, off, away; Gr. neut. n. *theion*, sulfur; Gr. masc. adj. *thermos*, hot; Gr. fem. n. *spora*, a seed; N.L. fem. n. *Dethiothermospor*a, a sulfur-reducing thermotolerant spore former).

Strictly anaerobic, motile, spore-forming rods that are thermotolerant and moderately halophilic. Capable of using thiosulfate and elemental sulfur as electron acceptors but not sulfide, nitrate or nitrite. Major fatty acids included anteiso-C15 : 0, C14 : 0 dimethyl acetyl, anteiso-C15 : 0 dimethyl acetyl, and iso-C15 : 0. DNA G+C content was 30.3 mol%. Whole-genome phylogeny locates *Dethiothermospora* in the family *Thermohalobacteraceae* within the order *Tissierellales* in the class *Tissierellia*. The type species is *D. halolimnae*.

## Description of *Dethiothermospora halolimnae* sp. nov.

*Dethiothermospora halolimnae* (ha.lo.lim’nae. Gr. masc. n. *hals*, salt; Gr. fem. n. *limne*, lake; N.L. gen. n. *halolimnae*, of a salt-lake).

Cells are Gram-stain-negative and are strictly anaerobic rods (~0.5×0.5 µm to 6 µm) occurring singly or in pairs, forming spores in older cultures. Cells are motile, but flagella were not observed. Fatty acids detected at levels above 2% in strain SD1^T^ included anteiso-C15 : 0 (24.9%), C14 : 0 DMA (13.2%), anteiso-C15 : 0 DMA (11.5%), iso-C15 : 0 (10.4%), C14 : 0 (7.4%), C14 : 0 aldehyde (7.2%), iso-C15 : 0 DMA (6.0%), iso-C14 : 0 (4%), C16 : 0 DMA (2.4%) and iso-C15 : 0 ALDE (2.4%) (Table 2). Growth occurs between 15 and 50 °C (optimum 40 °C), pH 6.0–10.0 (optimum 9.0) and in 1–150 g l^–1^ sodium chloride (optimum 50 g l^–1^). Able to grow on glycine and arginine, but not on cysteine, serine, lysine, valine, fructose, galactose, glucose, glycerol, mannitol, fumarate, cellobiose or cellulose. Capable of using elemental sulfur and thiosulfate as electron acceptors but not nitrate, nitrite, sulfate or sulfide. The type strain is SD1^T^ (= DSM 117405^T^ = TSD-443^T^), and it was isolated from the anaerobic porewater of a brine lake, Lake Koorkoordine, in the Yilgarn Craton, Western Australia. The DNA G+C content of the type strain is 30.3 mol%. The GenBank accession number of the whole-genome sequence and 16S rRNA gene sequence of the type strain SD1^T^ is GCA_037577855.1 and PP133767.1, respectively.

## Supplementary material

10.1099/ijsem.0.006760 Supplementary Material 1.

10.1099/ijsem.0.006760 Supplementary Material 2.

10.1099/ijsem.0.006760 Uncited Supplementary Material 3.

## Abbreviations

AAI: average amino acid identity
AFM: atomic force microscope
ANI: average nucleotide identity
DL: d-type low salt
GTDB: Genome Taxonomy Database
iTOL: interactive tree of life v7
NCBI: National Center for Biotechnology Information
POCP: percent of conserved protein
TDS: total dissolved solids
