# Supplementary material for: Dethiothermospora halolimnae gen. nov., sp. nov., a novel moderately halophilic, thermotolerant, bacterium isolated from a brine lake
Source: Int J Syst Evol Microbiol. 2025 Apr 30;75(4):006760. doi: 10.1099/ijsem.0.006760 (PMC12044194; doi:10.1099/ijsem.0.006760)
Supplement: Uncited Supplementary Material 3. [file ijsem-75-06760-s003.pdf]

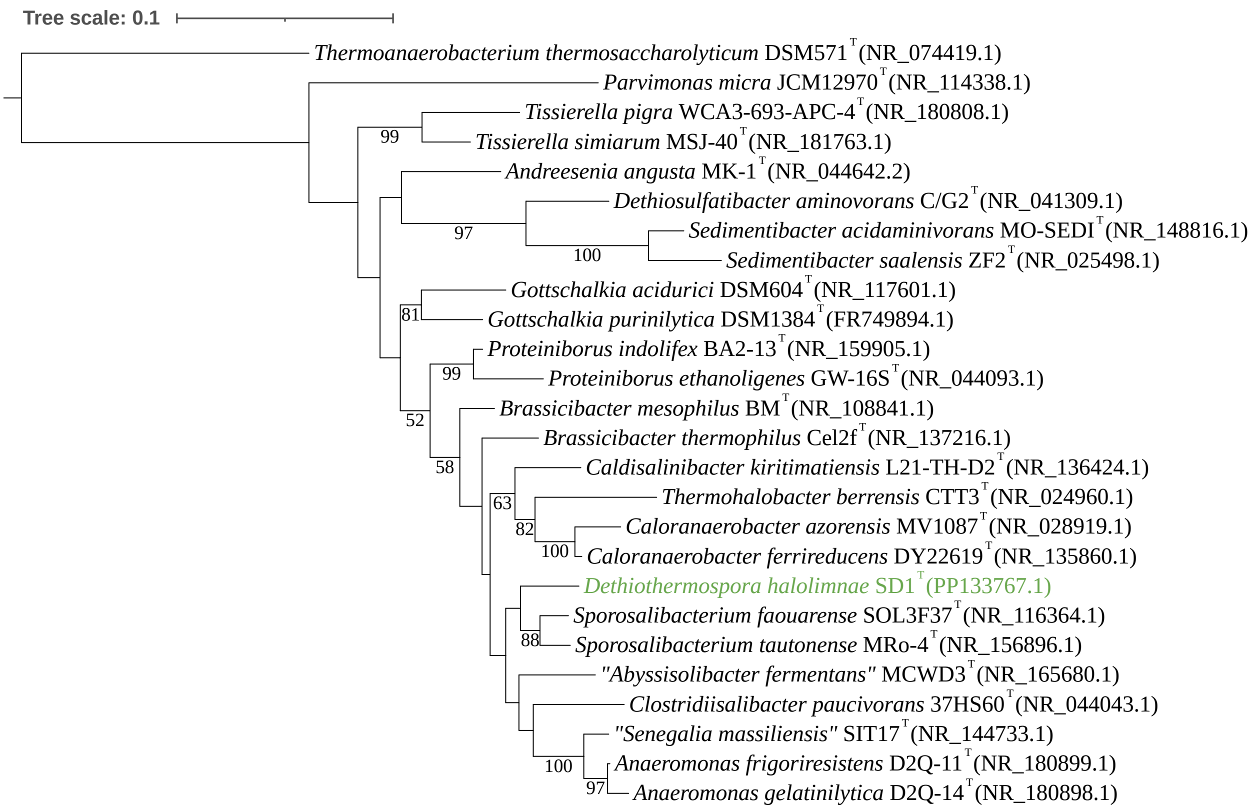

Supplementary Figure 1: Phylogenetic tree based on the 16S rRNA gene sequence of SD1^T (green) and close neighbors with *Thermoanaerobacterium thermosaccharolyticum* DSM 571 (accession number NR_074419.1) as an outgroup. 16S rRNA sequences were downloaded from NCBI, aligned with MAFFT v7.551 and trimmed using gBlocks v0.91b. The maximum-likelihood phylogenetic tree was constructed from the trimmed alignment using RAxML v8.2.12 using the GTR+G model and configured on ITOL. Bootstrap values (expressed as a percentage of 1,000 replicates) greater than 50 are indicated at nodes. Scale refers to the number of nucleotide substitutions per site between taxa.

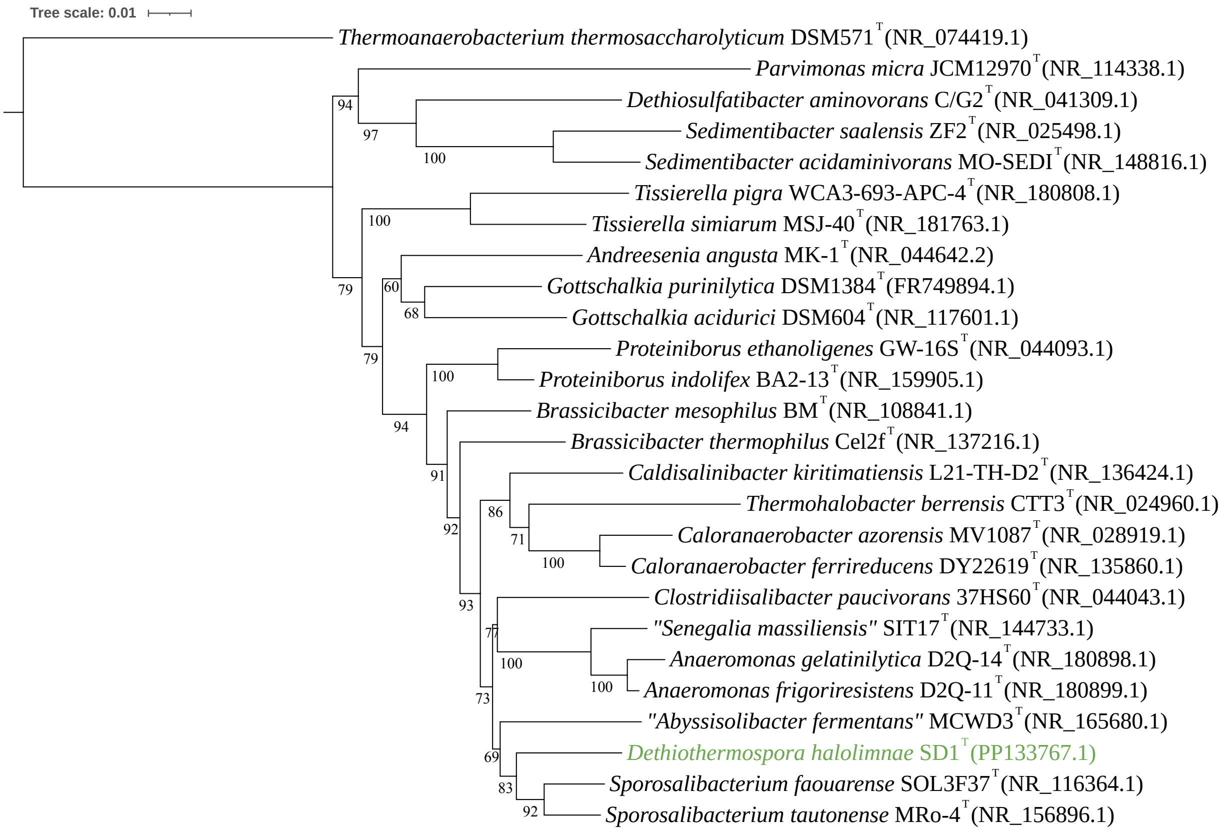

Supplementary Figure 2: Phylogenetic tree based on the 16S rRNA gene sequence of SD1^T (green) and close neighbors with *Thermoanaerobacterium thermosaccharolyticum* DSM 571 (accession number NR_074419.1) as an outgroup. 16S rRNA sequences were downloaded from NCBI, aligned with MAFFT v7.551 and trimmed using gBlocks v0.91b. The phylogenetic tree was constructed from the trimmed alignment using FastME 2.1.6.1.1. The Kimura two-parameter (K2P) model was used with Nearest Neighbor Interchanges (NNI) and Subtree Pruning and Regrafting (SPR) for tree refinement. Bootstrap values (expressed as a percentage of 1,000 replicates) greater than 60 are indicated at nodes. The tree was configured on ITOL, and scale refers to the number of nucleotide substitutions per site between taxa.

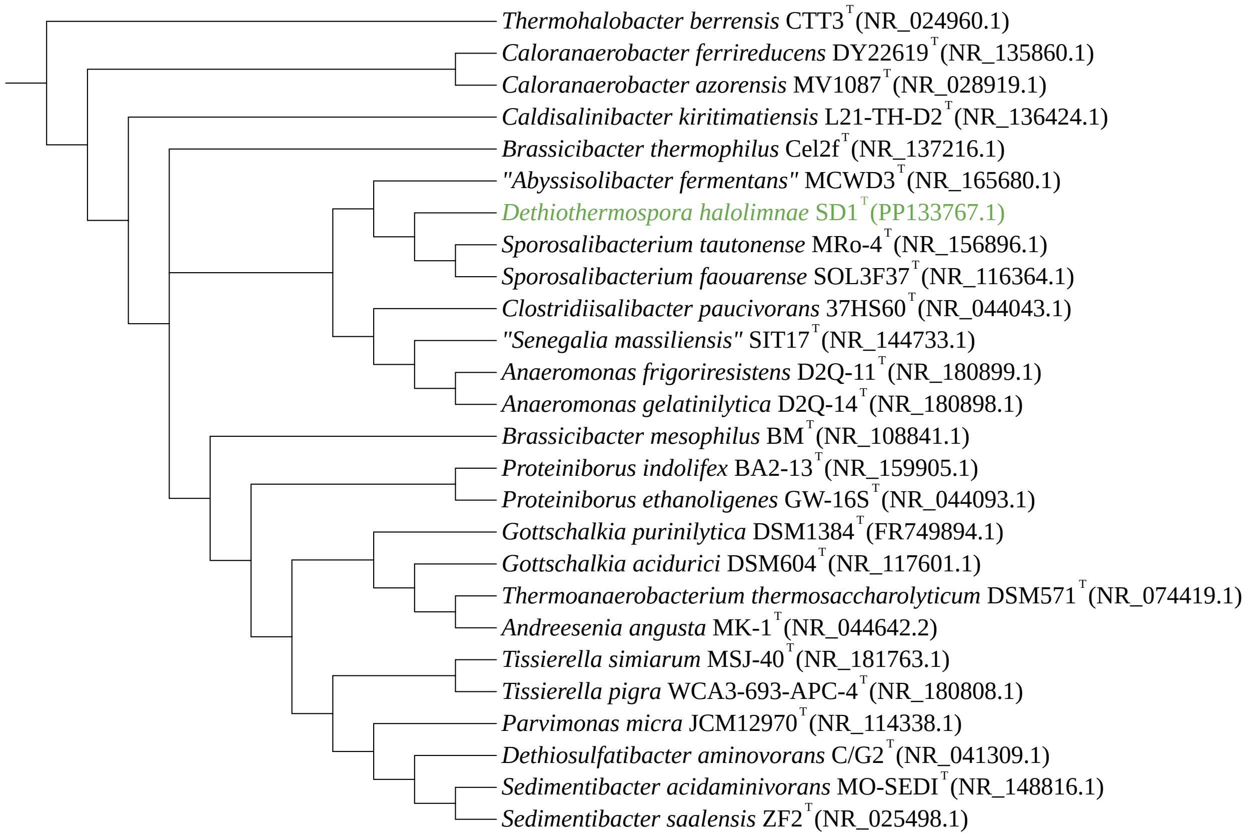

Supplementary Figure 3: A phylogenetic tree based on the 16S rRNA gene sequence of SD1^{T} (green) and close neighbors with *Thermoanaerobacterium thermosaccharolyticum* DSM 571 (accession number NR_074419.1) as an outgroup. 16S rRNA sequences were downloaded from NCBI, aligned with MAFFT v7.551 and trimmed using gBlocks v0.91b. The phylogenetic tree was constructed using maximum parsimony from the trimmed alignment using TNT 2.1.6.1.1 and configured on ITOL.

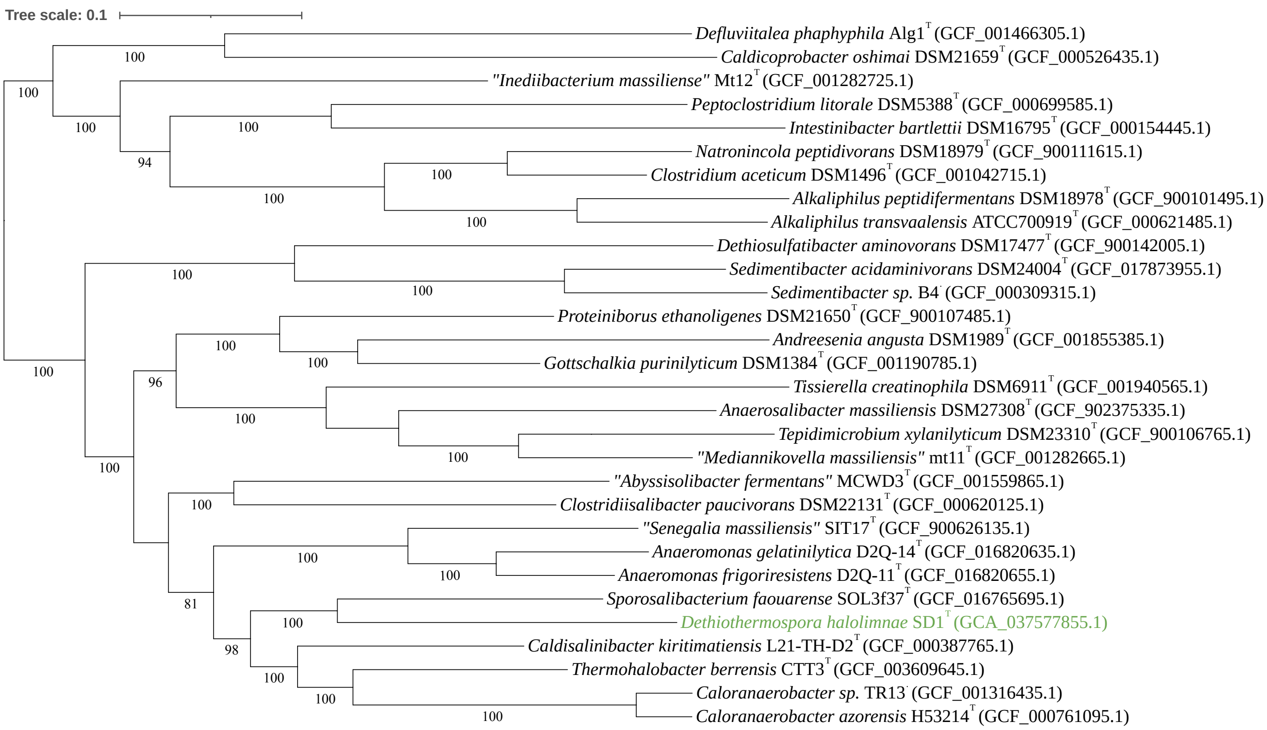

Supplementary Figure 4: A phylogenomic tree of strain SD1^{T} (green) in comparison with various publicly available RefSeq genomes of close neighbors. The tree was constructed using the Insert Genome into SpeciesTree application (v2.2.0) on KBase using FastTree (v2.1.10) default settings. Bootstrap values at nodes (expressed as a percentage of 1,000 replicates) above 80 are shown representing local support values computed using the Shimodaira-Hasegawa (SH) test. The tree was configured on ITOL, and scale refers to the number of nucleotide substitutions per site between taxa.

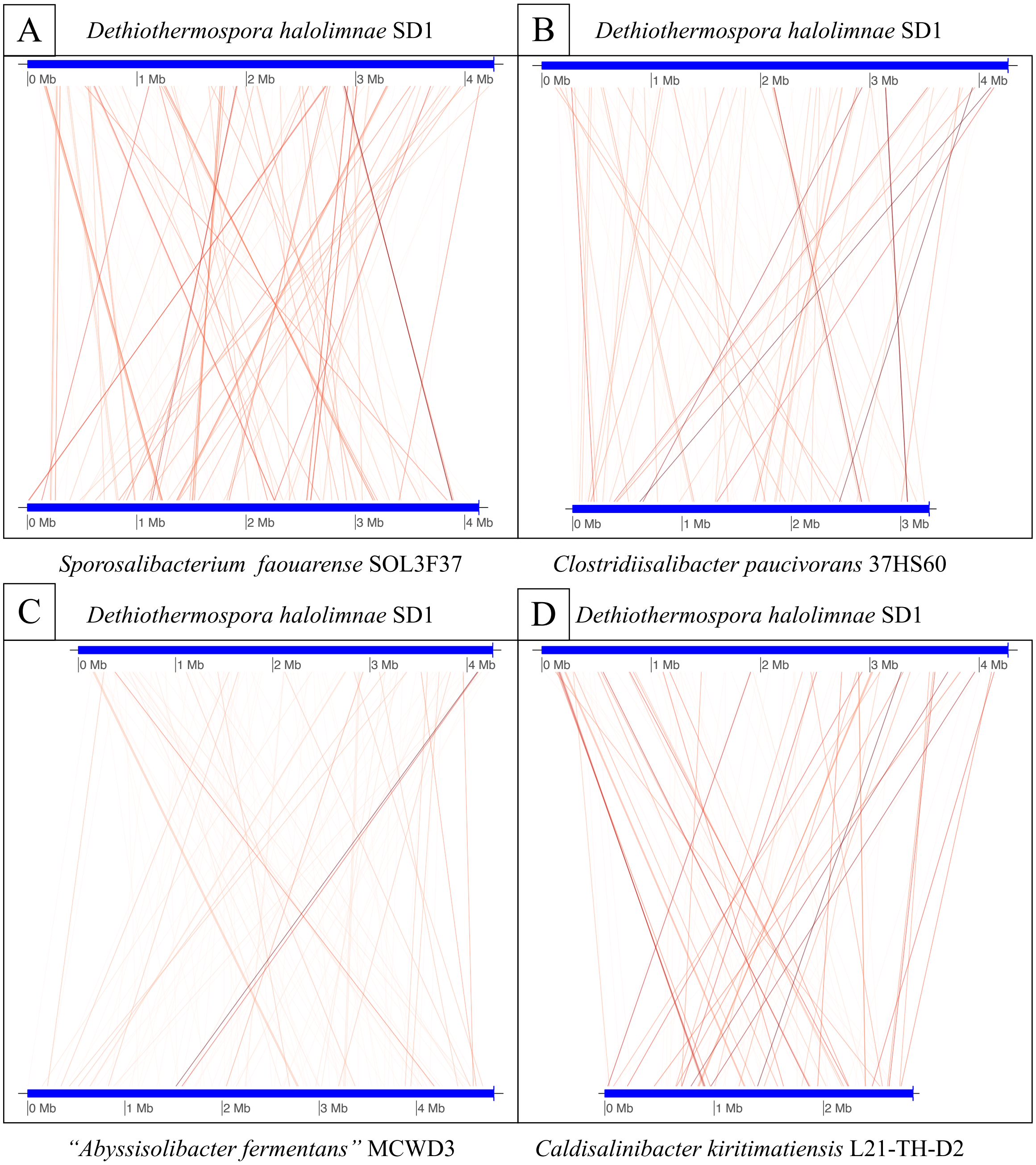

Supplementary Figure 5: Whole genome comparison of *Dethiothermospora halolimnae* SD1^T and its close neighbors (A) *Sporosalibacterium faourense* SOL3f37^T (76.31% ANI), (B) *Clostridiisalibacter paucivorans* 37HS60^T (76.18% ANI), (C) "*Abyssisolibacter fermentans*" MCWD3^T (75.68%), and (D) *Caldisalinibacter kiritimatiensis* L21-TH-D2^T (76.01%) using FastANI. Lines indicate reciprocal mapping among the two genomes showing conserved regions.
